# Complete laparoscopic ileal augmentation cystoplasty with modified ureteral reimplantation using an extra peritoneal approach for neurogenic bladder with vesicoureteral reflux in patient: a case report

**DOI:** 10.3389/fsurg.2025.1496767

**Published:** 2025-09-24

**Authors:** Qingfei Xing, Li He, Peiyan Jin, Dongxu Tian, Ke Wang, Feng Guo

**Affiliations:** ^1^ Department of Urology, Central Hospital Affiliated to Shandong First Medical University, Jinan, Shandong, China; ^2^ Department of Gastroenterology, Shandong Province Hospital, Jinan, Shandong, China; ^3^ Department of Urology, Affiliated Hospital of Qingdao University, Qingdao, Shandong, China

**Keywords:** neurogenic bladder, vesicoureteral refux, augmentation cystoplasty, extraperitoneal, complete laparoscopic

## Abstract

**Background:**

Neurogenic bladder (NGB) is defined as bladder dysfunction. Patients with NGB often have issues with high-pressure storage of urine in the bladder and/or coordinated emptying of urine. Neurogenic bladders with high pressure may lead to vesicoureteral reflux (VUR).High-grade VUR leads to recurrent urinary tract infections (UTIs), and thus causes severe renal failure. Augmentation cystoplasty (AC) can reduce bladder pressure, increase bladder compliance, reduce vesicoureteral reflux, reduce the incidence of urinary incontinence, and improve the quality of life of patients. In recent years, with the maturity of laparoscopic technology, laparoscopic ileal augmentation cystoplasty has been widely used in clinical practice. However, through searching the database, we did not find any medical records of AC performed under complete laparoscopic via extraperitoneal approach. We report today a case of complete laparoscopic ileal augmentation cystoplasty with modified ureteral reimplantation using an extraperitoneal approach for neurogenic bladder with vesicoureteral reflux in a patient.

**Case presentation:**

A 61-year-old women was hospitalized with symptoms of recurrent frequent urination. The patient had a urine volume of about 50 ml each time, no symptoms of dysuria, and no previous history of tuberculosis, diabetes and lumbar disc herniation. After admission, in addition to routine examinations, we also conducted abdominal CT, retrograde cystography, cystoscopy and urodynamic examination for the patient. Eventually, the patient was diagnosed with neurogenic bladder and bilateral ureteral reflux. The patient underwent a complete laparoscopic ileal augmentation cystoplasty with modified ureteral reimplantation using an extraperitoneal approach. Six months after the operation, the bladder volume expanded to 400 ml, and acute pyelonephritis did not occur. We ordered a CT scan of the patient, which showed no dilated ureter.

**Conclusion:**

Complete laparoscopic ileal augmentation cystoplasty with modified ureteral reimplantation using an extraperitoneal approach is difcult due to the complex operation procedure and technical difculties. This investigation demonstrated that the extraperitoneal technique of enterocystoplasty that we describe is safe and feasible and has the advantages of less trauma, less bleeding, faster return of intestinal function, and fewer postoperative complications.

## 1 Introduction

The elasticity and neuromodulation of the detrusor muscle maintain good compliance and stability of the bladder, thereby maintaining low pressure in the bladder for storage of urine. Neurogenic bladder (NGB) is defined as bladder dysfunction due to neurologic conditions that may afect the central or peripheral nervous system. Patients with NGB often have issues with high-pressure storage of urine in the bladder and/or coordinated emptying of urine (1). Neurogenic bladders with high pressure may lead to vesicoureteral refux (VUR). High-grade VUR leads to recurrent urinary tract infections (UTIs), and thus causes severe renal failure.

Initially, as conservative treatment, clean intermittent catheterization (CIC) and anticholinergic therapy were generally conducted. Intravesical injection of botulinum toxin A is also efective to improve bladder compliance and capacity. If these conservative managements are inefective, augmentation cystoplasty (AC) is generally accepted as one of the standard therapeutic options (2). AC can reduce bladder pressure, increase bladder compliance, reduce vesicoureteral reflux, reduce the incidence of urinary incontinence, and improve the quality of life of patients (3–5).

AC is a type of reconstructive surgery in which it is usually done with a section of the ileum or colon that is separated from the rest of the gastrointestinal tract and relies on mesenteric blood supply. Further techniques include detubularization or reconfiguration as needed, where the bladder is opened to fit this section of bowel into the bladder.The purpose of the surgery is to expand the volume of the bladder and decrease its pressure and spasticity (6).

Mortality rates for AC range between 0% and 2.7%, the rates of early morbidity are also significant (7).Complications of AC: intestinal obstruction,urinary tract infection,bladder stones,metabolic disorders and bladder rupture, etc.Intraperitoneal bladder rupture occurs in 0.8%–13% of cases and can be life-threatening, as the urine from the reconstructed bladder contains bacteria that can lead to widespread contamination of the peritoneal cavity when ruptured (8, 9). About 10% of patients who underwent AC with a minimum of 20 years followup developed an small bowel obstruction (SBO), with half of those patients requiring exploratory laparotomy (10).

Given the consequences and severity of a ruptured bladder and SBO and its resultant complications, we potentially minimize this risk after AC by extraperitonealizing the bladder augmentation.

In the past, open surgery was often used for AC (11). In recent years, with the maturity of laparoscopic technology, laparoscopic ileal augmentation cystoplasty has been widely used in clinical practice (12, 13). However, through searching the database, we did not find any medical records of AC performed under complete laparoscopic via an extra peritoneal approach. We report today a case of complete laparoscopic ileal augmentation cystoplasty with modified ureteral reimplantation using an extra peritoneal approach for neurogenic bladder with vesicoureteral reflux in a patient.

## 2 Case presentation

**Figure 1:**
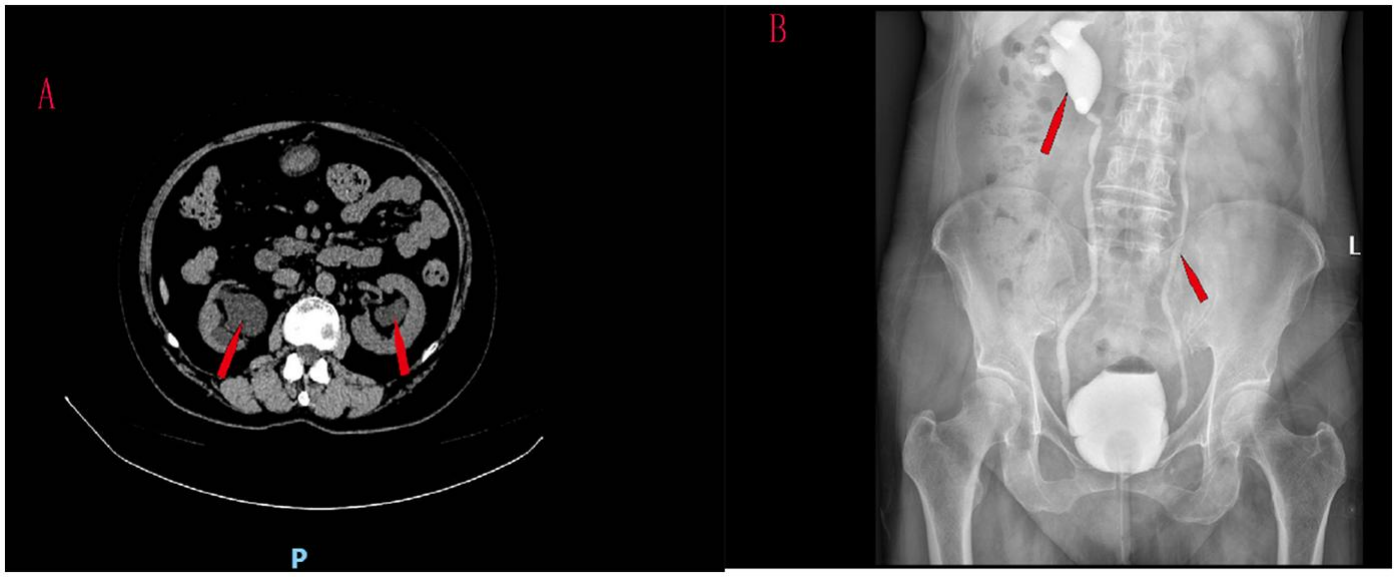
CT scan showed bilateral ureteral hydrodilation **(A)** reflux grade was V in the right ureter and III in the left ureter **(B)**.

A 61-year-old women was hospitalized with symptoms of recurrent frequent urination, urgent urination and odynuria accompanied by fever and right lumbar pain. The patient had a urine volume of about 50 ml each time, no symptoms of dysuria, and no previous history of tuberculosis, diabetes and lumbar disc herniation. CT scan showed bilateral ureteral hydrodilation (Figure 1A). Preoperative blood creatinine was 167 μmol/L. Tests for tuberculosis were negative. The patient underwent magnetic resonance imaging (MRI) examinations of the brain and spinal cord, and no neurological lesions were found.

Cystography was performed. Vesicoureteral reflux was observed when 80 ml of contrast media was injected into the bladder, and the patient developed a strong urge to urinate. VUR was found in this patient, involving two ureters. Reflux grade was V in the right ureter and III in the left ureter (Figure 1B).

Urodynamic examination suggested a neurogenic bladder. During the imaging urodynamic examination, 22.7 ml of contrast media was injected into the bladder through a catheter. The patient experienced a strong urge to urate. At this time, the detrusor pressure was 17.27 cmH2O. When the maximum bladder capacity was 59.8 ml, the detrusor pressure was 31.25 cmH2O, and at the time, bilateral ureteral reflux occurred. Cystoscopy was performed on the patient, which revealed bladder diverticulum, and no obvious obstruction of the bladder outlet. The patient underwent a complete laparoscopic ileal augmentation cystoplasty with modified ureteral reimplantation using an extraperitoneal approach.

## 3 Therapeutic process

**Figure 2:**
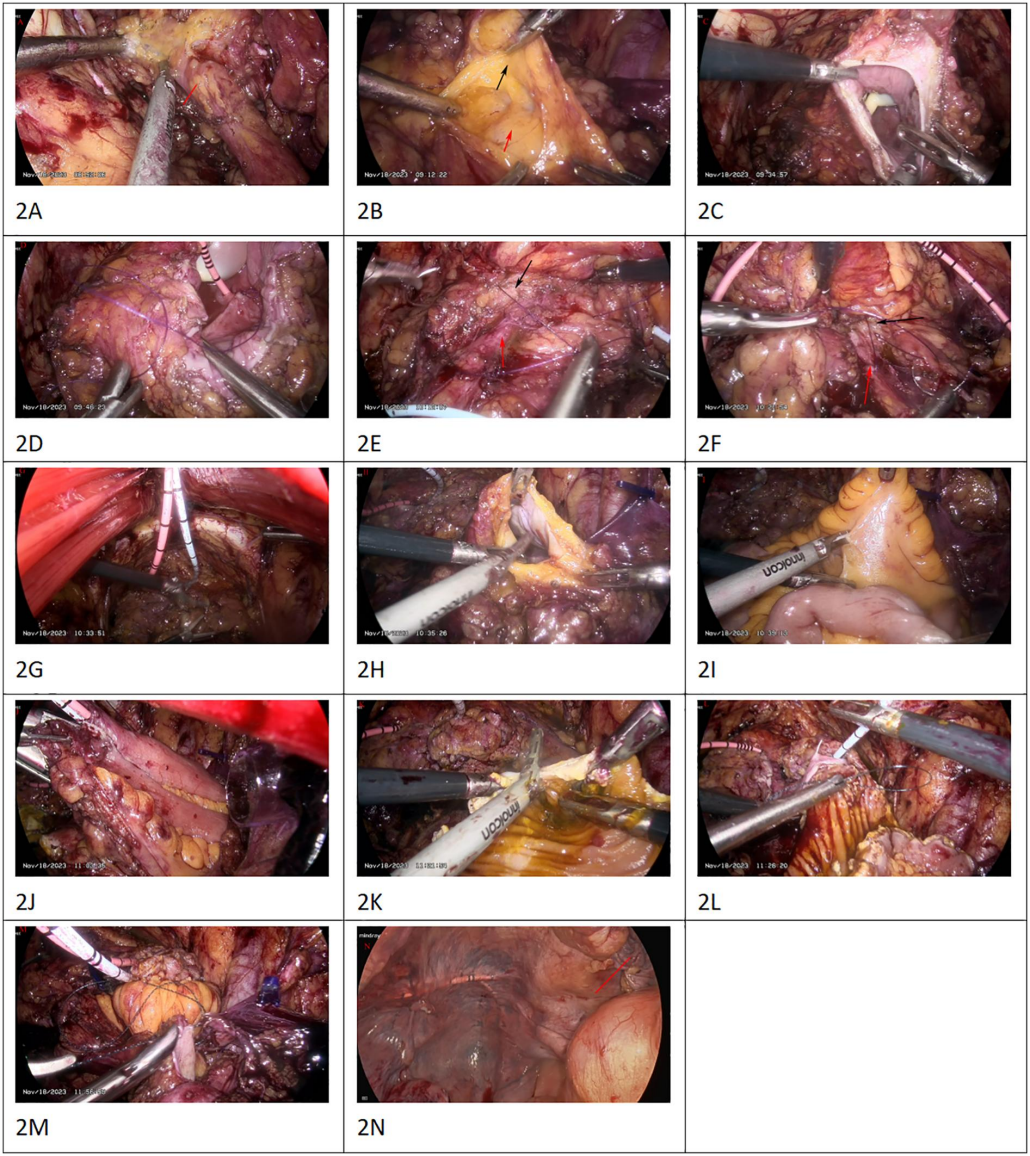
The peritoneum was gently pushed inward to free the ureter [**(A)**, red arrow]. The peritoneum is carefully dissected off the bladder both sharply and bluntly.The peritoneum covering the top and posterior walls of the bladder was swam away [**(B)**, the red arrow is peritoneum. The black arrow is the wall of the bladder]. Video 1 the apex of the bladder wall was excised horizontally **(C)** The single J stent was inserted into the ureter and drained from the abdominal wall and fixed. We used 3-0 absorbable sutures to narrow the bilateral ureteral orifices and free the ureteral end. After folding the ureteral end with 3-0 absorbable sutures, the ureteral end was embedded with the myometrium of the bladder. [**(D–G)**; the red arrow is ureter. The black arrow is the myometrium of the bladderv. Video 2–5 the peritoneum was cut 4 cm long above the right iliac blood duct **(H)** Under the laparoscopic light source, about 15 cm from the ileocecal region, the terminal ileum was removed for 20 cm **(I)**. A lateral anastomosis of the distal and proximal ileum was performed using a linear closure (EC60 stapler) to restore intestinal continuity **(J)**. After irrigation with iodine solution, the intestinal wall was longitudinally incised at the opposite side of the mesentery. During the operation, the intestinal wall on the opposite side of the mesentery was incised as far as possible along the midline **(K)**. The intestinal segment was sutured to the apical wall of the bladder with absorbable suture **(L)**. The peritoneum was sutured by simple continuous absorbable thread around the intestinal loop mesentery **(M)**. The trocar was inserted into the abdominal cavity to examine the bowel and the peritoneum was well closed [**(N)**, red arrow].

Routine bowel preparation was performed the day before surgery. After anesthesia took effect, the patient was placed in a 15° Trendelenburg position. A five-point puncture channel was used. An infraumbilical midline incision about 4 cm long is made into retropubic space. A balloon dilator was inserted into retropubic space to expand the extraperitoneal space. The 12 mm trocars were inserted through the right and left rectus abdominis, 6 cm below the umbilicus. Artificial pneumoperitoneum was established, and 5 mm trocars at 3 cm above the left and right anterior suprailiac spine, respectively. The peritoneum is then carefully dissected off the bladder both sharply and bluntly. It is easier to initiate the dissection on the lateral aspect. The space between the peritoneum and the external iliac blood vessel was separated along the neck of the bladder. The space between the iliac fossa was expanded. The peritoneum was gently pushed inward to free the ureter (Figure 2A). The space of adipose tissue outside the bladder and the peritoneal was expanded. The peritoneum covering the top and posterior walls of the bladder was swam away (Figure 2B; Video 1).At this point, the bladder is completely freed off the intact peritoneum. Then, 100 ml of water was injected into the bladder via a urethral catheter, and the apex of the bladder wall was excised horizontally (Figure 2C). The patient with vesicoureteral reflux underwent modified laparoscopic ureterovesical reimplantation at the same time. The single J stent was inserted into the ureter and drained from the abdominal wall and fixed. We used 3-0 absorbable sutures to narrow the bilateral ureteral orifices and free the ureteral end. The distal end of the ureter was fully released into the inner segment of the ureter wall. After folding the ureteral end with 3-0 absorbable sutures.The end of the ureter was buried in the bladder myometrium with 3-0 absorbable sutures. (Figure 2D–G; Video 2–5) The peritoneum was cut 4 cm long above the right iliac blood duct (Figure 2H).

Under the laparoscopic light source, about 15 cm from the ileocecal region, the terminal ileum was removed for 20 cm. (Figure 2I) A lateral anastomosis of the distal and proximal ileum was performed using a linear closure (EC60 stapler) to restore intestinal continuity (Figure 2J). 3-0 absorbable sutures were used to close the mesenteric laceration. When suturing the mesenteric laceration, care should be taken not to tighten too tightly to avoid affecting the blood supply of the ileal loops. The ileum was returned to the abdominal cavity.

After irrigation with iodine solution, the intestinal wall was longitudinally incised at the opposite side of the mesentery. During the operation, the intestinal wall on the opposite side of the mesentery was incised as far as possible along the midline (Figure 2K). The intestinal segment was sutured to the apical wall of the bladder with absorbable suture (Figure 2L).

The intestinal tract was inserted into the peritoneal cavity, intestinal loop was incorporated into the extraperitoneal pelvic cavity, and the peritoneum was sutured by simple continuous absorbable thread around the intestinal loop mesentery (Figure 2M). This allows for an adequate blood supply while keeping the opening small enough to prevent herniation. Injection of water into the bladder did not reveal any obvious leakage of urine. A drainage tube was placed in the pelvic cavity and the operation was completed. The trocar was inserted into the abdominal cavity to examine the bowel and the peritoneum was well closed (Figure 2N).

### 3.1 Follow-up and prognosis

**Figure 3:**
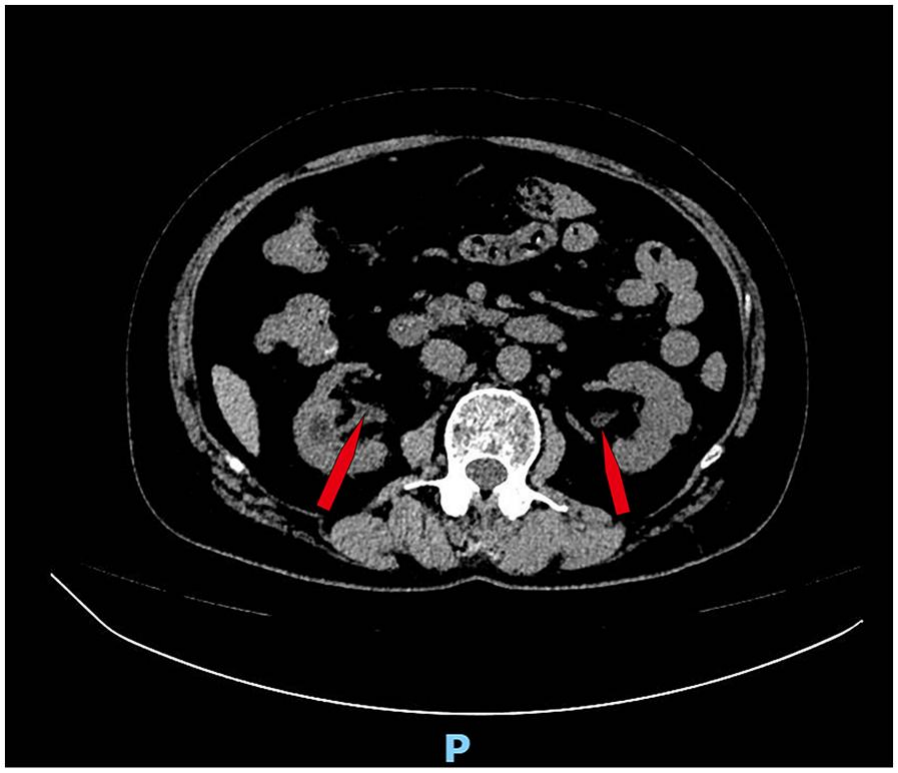
CT scan showed no dilated ureter (Figure 3, red arrow).

Six months after the operation, the patient returned to the hospital for follow-up review, the creatinine level decreased to normal, the bladder volume expanded to 400 ml, and acute pyelonephritis did not occur. Because of concerns about the risk of urinary tract infection, cystography was not performed to determine whether the vesicoureteral reflux was improved. We ordered a CT scan of the patient, which showed no dilated ureter (Figure 3).

## 4 Discussion

Neurogenic bladder is mainly due to the patient's central or peripheral nerve regulating urination damage, resulting in reduced bladder compliance, and then reduced bladder safety capacity, resulting in increased bladder pressure, and eventually lead to vesicoureteral reflux, affecting the patient's kidney function. The principle of treatment is to protect kidney function and thus improve the quality of life of the patient. In the treatment of low bladder compliance, measures that can effectively reduce pressure in the bladder include suprapubic vesical fistula, clean intermittent self-catheterization, catheterization, and various types of AC (14, 15).

According to the European Urology Association (EUA) guidelines on neurourology, AC is suitable for patients who have failed conservative treatment (16). These conservative measures include medications such as anticholinergics and minimally invasive treatments such as intravesical botulinum toxin injection and, in rare cases, sacral neuromodulation.

AC is currently the gold standard surgery to increase bladder capacity, decrease intravesical storage pressure, and improve upper urinary tract drainage (17). Due to the difculty of ileal AC and its complex steps, open surgery has predominantly been used in the past, however, open surgery is more traumatic to patients and has more postoperative intestinal complications (11).

In recent years, with the widespread use of laparoscopic techniques, more and more minimally invasive and effective methods have been studied in clinical practice. There have been more and more reports about the use of AC with laparoscopic or robot-assisted laparoscopic (12, 13). Complications of AC: intestinal obstruction, urinary tract infection, bladder stones, metabolic disorders and bladder rupture, etc. Reyblat et al. conducted a retrospective chart review of 73 patients who underwent an extraperitoneal (*n* = 49) vs. intraperitoneal (*n* = 24) bladder augmentation approach and discovered that complication rates (such as rates of stones, ileus,infection, bowel obstruction, and wound dehiscence) also did not differ (7). When comparing the intraperitoneal group with the extraperitoneal group, Reyblat found a significantly shorter operative time, less blood loss, sooner return of bowel function, and shorter hospital stay associated with the extraperitoneal group (7).

In order to reduce postoperative complications such as abdominal infection caused by bladder rupture, intestinal obstruction and accelerate postoperative recovery, we performed a complete laparoscopic ileal augmentation cystoplasty with ureteral reimplantation using an extraperitoneal approach for the patient. Complete laparoscopic ileal enhanced cystoplasty can reduce the effect of surgery on intestinal function. Laparoscopy provides a clear enlarged field of view, helps to identify the mesenteric blood vessels, and reduces the damage to the intestinal blood supply, which is conducive to the recovery of intestinal function. Complete laparoscopic surgery can prevent intestinal exposure, reduce intestinal interference, and reduce postoperative intestinal adhesion and intestinal adhesion obstruction.

Neurogenic bladders with high pressure may lead to VUR, which can cause pyelonephritis, renal scarring, and renal insufficiency. Whether ureteral reimplantation should be done simultaneously with AC has been controversial. After ileal augmentation cystoplasty, the capacity of the bladder is increased and pressure is decreased, and in some cases ureteral refux can disappear or improve spontaneously. The majority of patients with VUR who undergo AC will have resolution of their VUR following surgery, which potentially precludes the need for concomitant ureteral reimplantation (18, 19). Previous studies have shown that ureteral replantation should be performed in patients with low or high-grade VUR, obstruction of the ureterovesical junction, and severe upper urinary tract dilation (20–23). In the patient we reported, cystography revealed grade V VUR in the right ureter and grade III VUR in the left ureter. Ureteral bladder replantation was performed on the patient. It is not easy to re-implant the ureter into the thick bladder wall, so the ureter needs to be implanted into the ileum wall during the operation. However, due to the thin ileum wall and poor resistance to reflux, the tunnel method is not effective. The nipple method is easy to fit and has good reflux resistance, especially suitable for ileum enhanced cystoplasty (24). Because ureteral replantation is currently controversial, and to avoid postoperative ureteral stricture, we used a modified ureteral bladder replantation. Postoperative CT examination showed that the right ureter had no dilated hydrops, and the renal function returned to normal.

## 5 Conclusion

The operation procedure of complete laparoscopic ileal augmentation cystoplasty with modified ureteral reimplantation using an extraperitoneal approach is complex. This preliminary investigation demonstrated that the extraperitoneal technique of enterocystoplasty that we describe is feasible and has the advantages of less trauma, less bleeding, faster return of intestinal function, and fewer postoperative complications.

## Data Availability

The original contributions presented in the study are included in the article/Supplementary Material, further inquiries can be directed to the corresponding authors.
